# Cellular interactions between L-arginine and asymmetric dimethylarginine: Transport and metabolism

**DOI:** 10.1371/journal.pone.0178710

**Published:** 2017-05-31

**Authors:** Soyoung Shin, Subindra Kazi Thapa, Ho-Leung Fung

**Affiliations:** 1 Department of Pharmacy, College of Pharmacy, Wonkwang University, Iksan, Jeonbuk, Korea; 2 Department of Pharmaceutical Sciences, University at Buffalo, The State University of New York, Buffalo, New York, United States of America; Augusta University, UNITED STATES

## Abstract

This study was aimed to examine the effect of L-arginine (ARG) exposure on the disposition of asymmetric dimethylarginine (ADMA) in human endothelial cells. Although the role of ADMA as an inhibitor of endothelial nitric oxide synthase (eNOS) is well-recognized, cellular interactions between ARG and ADMA are not well-characterized. EA.hy926 human vascular endothelial cells were exposed to ^15^N_4_-ARG, and the concentrations of ^15^N_4_-ARG and ADMA in the cell lysate and incubation medium were determined by a liquid chromatography-electrospray tandem mass spectrometry (LC-MS/MS) assay. Nitric oxide (NO) production was estimated by utilizing cumulative nitrite concentration via a fluorometric assay. Cells incubated with ^15^N_4_-ARG exhibited enhanced nitrite production as well as ^15^N_4_-ARG cellular uptake. These changes were accompanied by a decrease in cellular ADMA level and increase in extracellular ADMA level, indicating an efflux of endogenous ADMA from the cell. The time courses of ADMA efflux as well as nitrite accumulation in parallel with ^15^N_4_-ARG uptake were characterized. Following preincubation with ^15^N_4_-ARG and D_7_-ADMA, the efflux of cellular ^15^N_4_-ARG and D_7_-ADMA was significantly stimulated by high concentrations of ARG or ADMA in the incubation medium, demonstrating trans-stimulated cellular transport of these two amino acids. D_7_-ADMA metabolism was inhibited in the presence of added ARG. These results demonstrated that in addition to an interaction at the level of eNOS, ARG and ADMA may mutually influence their cellular availability via transport and metabolic interactions.

## Introduction

The use of L-arginine (ARG) as a dietary supplement for improving the conditions of patients with a variety of diseases, including congestive heart failure, intermittent claudication, bladder inflammation, wasting and weight loss in people with HIV/AIDS, and erectile dysfunction has been extensively investigated [1]. These effects occur principally because ARG serves as a substrate for various nitric oxide synthases (NOS) to produce nitric oxide (NO) and L-citrulline (CIT). NO is an endogenous endothelial vasodilator, and its enhanced production is beneficial in diseases that are associated with endothelial dysfunction.

Although short-term ARG supplementation has been proven to improve NO-mediated vascular function, it is uncertain as to why it works at all. The intracellular concentration of endogenous ARG is typically > 1 mM [2], while the Michaelis-Menten Km of endothelial NOS, determined for the isolated and purified enzyme for ARG in vitro, is about 3 μM [3]. Thus, minor increases in extracellular ARG concentration, derived from supplementation, would not be expected to cause considerably enhanced NO production, since the Km of the enzyme is already vastly exceeded by the resident intracellular ARG concentration. This puzzle has been termed the “L-arginine paradox.”

One of the widely accepted hypotheses to explain this phenomenon involves the role of an endogenous inhibitor of NOS, asymmetric dimethylarginine (ADMA, Ki = 0.9 μM) [4, 5]. This hypothesis posits that endogenous ADMA increases the apparent Km of NOS for ARG in vivo, and supplementation with ARG displaces ADMA from NOS, resulting in enhanced NO activity [6, 7].

A major concern about the validity of this hypothesis is that the mean circulating ADMA concentration in plasma is quite low, of about 0.5 μM, and kinetic analysis of its displacement from the enzyme active site would not be sufficient to explain the effects of ARG supplementation. However, the interaction of ADMA with ARG should not be restricted to the endothelial NOS (eNOS) enzyme only, because other cellular processes, such as those involving cellular transport and metabolism, could also be altered at different cellular ADMA/ARG concentration ratios.

The cellular uptake of ARG is mediated predominantly (70–95%) via the cationic amino acid transporter 1 (CAT-1), which belongs to the system y+ family of cationic amino acid transporters [8, 9]. These transporters possess common characteristics such as Na^+^ independence, pH insensitivity, and strong stimulation by substrate on the opposite side of the membrane barrier (trans-stimulation), which mediates the transmembrane exchange of extracellular and intracellular amino acids [10]. It has been shown that ADMA exerts an inhibitory effect on ARG transport [11, 12]. Using ^15^N_4_-ARG as a probe, we have also reported that endogenous cellular ARG and ADMA efflux was induced by exogenous ARG exposure [13]. However, the net effect of ARG exposure on both ARG and ADMA influxes and effluxes has not been examined.

Systemic ADMA elimination occurs primarily (>70%) through metabolism wherein the intracellular enzyme dimethylarginine dimethylaminohydrolase (DDAH) forms dimethylamine and CIT [14–16] and a small amount of ADMA is excreted into the urine. Decreased DDAH expression and activity are evident in diseases associated with endothelial dysfunction, and are believed to underlie the mechanism responsible for increased ADMA and impaired NO production [17–20]. It has been reported that ARG can inhibit DDAH and raise cellular ADMA concentration in HepG2 cells [21]. However, it is not known whether this metabolic interaction between ARG and ADMA is operative in physiologically relevant vascular endothelial cells.

Therefore, in the present study, we investigated the interactions between ADMA and ARG concerning their cellular transport and mutual displacement as well as metabolism.

## Materials and methods

### Chemicals and reagents

ARG [as L-arginine HCl], ADMA [as *N*^G^,*N*^G^-dimethylarginine dihydrochloride], and SDMA [as *N*^G^,*N*^G^′-dimethyl-L-arginine di(p-hydroxyazobenzene-p′-sulfonate) salt] were purchased from Sigma. Stable isotope-labeled compounds, ^15^N_4_-ARG [as its HCl, (U-^15^N_4_, 98%)], ^13^C_6_-ARG [as ARG:HCl (U-^13^C_6_, 98%)], and D_7_-ADMA [as ADMA:HCl:H_2_O (2,3,3,4,4,5,5-D7, 98%)] were obtained from Cambridge Isotope Laboratories, Inc. These compounds were used without further purification. Cell culture reagents were purchased from Invitrogen.

### Cell culture

The EA.hy926 human vascular endothelial cell line, which is a continuous cell line derived from human umbilical vein endothelial cells [22], was grown in Dulbecco’s modified Eagle’s medium (DMEM) with 2 mM glutamine supplemented with 10% fetal bovine serum, 100 U/mL penicillin, and 100 μg/mL streptomycin at 37°C in a 5% CO_2_ incubator [10]. Since concurrent work showed that high concentrations of glucose (4.5 G/L) and ARG (84 mg/L) in the original DMEM medium inhibited eNOS activity [23], a further-modified DMEM, which contained 0.9 G/L of glucose and 21 mg/L of ARG was used for ADMA metabolism studies. Cells were used for experiments after they reached confluence in 6-well plates in 7 days.

### Measurement of cellular fluxes of ADMA

The cells were washed and equilibrated in Locke’s solution (LS; 154 mM NaCl, 5.6 mM KCl, 2 mM CaCl_2_, 1 mM MgSO_4_, 10 mM 4-(2-hydroxyethyl)-1-piperazineethanesulfonic acid (HEPES), 3.6 mM NaHCO_3_ and 5.6 mM glucose) for 1 hour before treatment. Fresh LS containing ^15^N_4_-ARG was added and the cells were incubated at 37°C. After 2 hours, the cell incubation medium was collected, and cells were washed with ice-cold LS, and collected. Collected cells were resuspended in LS and lysed by sonication (10 s × 5). After centrifugation, the supernatant was collected for liquid chromatography-electrospray tandem mass spectrometry (LC-MS/MS) analysis. The protein concentration in the cell lysates was determined by the method of Lowry [24].

To examine trans-stimulation of ARG and ADMA, EA.hy926 cells were pretreated with the stable isotope-labeled compounds, i.e., ^15^N_4_-ARG (100 μM) or D_7_-ADMA (10 μM) for 1 hour, and washed 5 times with LS. These cells were then, exposed to ARG (1 mM) or ADMA (1 mM) added to the fresh medium. After 1 hour of exposure, the concentration of ^15^N_4_-ARG or D_7_-ADMA in the cell lysate and incubation medium was determined.

### Measurement of ARG-ADMA interaction in membrane fragments

Cell membrane fragments were prepared according to Zharikov et al. [25]. After cells were scraped into pre-cooled Hank’s balanced salt solution, they were centrifuged at 800 x g for 15 min. Then, cell pellets were resuspended in buffer A (0.25 M sucrose, 0.2 mM MgSO_4_, 10 mM HEPES-Tris, pH = 7.4), and centrifuged again at 800 x g. The resulting cell pellet was resuspended with buffer A at a pellet/buffer ratio of 1:9 (v/v), and the cells were disrupted by nitrogen cavitation using a pre-cooled mini cell disruptor with nitrogen pressure of 650 psi. After 15 min, the suspension was discharged dropwise from the instrument. The broken-cell suspension was then, centrifuged at 2000 x g for 5 min to remove nuclei, large aggregates, and broken cells. The postnuclear supernatant was collected, and centrifuged at 85000 x g for 20 min. The resulting pellet was resuspended in buffer A, and layered over a discontinuous sucrose gradient (15, 30, and 45%). The gradient was centrifuged at 100,000 x g for 1 hour at 4°C, and the bands at 15/30% sucrose interface were collected, and diluted with cold 10 mM HEPES-Tris (pH = 7.4). After centrifugation at 85,000 x *g* for 20 min, the final pellet of plasma membrane fragments was suspended in buffer A at a concentration of 3–5 mg of protein/mL, and stored at -80°C.

The study of transport in the membrane fragments was conducted as described in the literature [25, 26]. Membrane fragments in buffer A were rapidly thawed at 37°C, and diluted to 10 volumes of a loading solution containing 140 mM potassium phosphate (pH = 6.8) and 1 mM MgSO_4_. After incubation for 30 min at 4°C, the suspension was centrifuged at 48,000 x g for 15 min, and the pellet was resuspended in the loading solution to a final concentration of 2–3 mg protein/mL. A 30 μL aliquot of resuspended pellet solution was added to 470 μL of external solution containing 140 mM LiCl and 1 mM MgSO_4_ in 10 mM HEPES-Tris (pH = 7.4) and 100 μM of ^15^N_4_-ARG. After incubation in a shaking incubator at 37°C for 1 hour, the solutions were centrifuged at 48,000 x g for 15 min. The supernatant was collected, and the pellet was solubilized by adding 2% Triton X-100 and sonicated for analysis.

### Measurement of ADMA metabolism

The cells and cell lysates were washed and equilibrated in LS, and 100 μM D_7_-ADMA in LS was added. After 1 hour of incubation, the cells were washed, and fresh LS containing ^15^N_4_-ARG was added. After incubation, the incubation medium was collected. Cells were collected after trypsinization, and lysed by using 2% Triton-X-100 (lysis buffer). The concentrations of D_7_-ADMA, ADMA, ARG, and SDMA were determined by the LC-MS/MS assay.

### LC-MS/MS

Concentrations of ^15^N_4_-ARG, ARG, ADMA, SDMA, and D_7_-ADMA were determined by hydrophilic-interaction liquid chromatography-electrospray tandem mass spectrometry (LC-MS/MS) [13]. Briefly, samples were mixed with the internal standard (IS), ^13^C_6_-ARG 1 μM, and mobile phase B (acetonitrile containing 0.025% trifluoroacetic acid (TFA), and 0.5% acetic acid) was added to the sample for protein precipitation. Precipitated protein was then, removed by centrifugation at 10,000 × g for 20 min. Analytes were separated by liquid chromatography on a 150 × 2.1 mm Alltima HP HILIC 3μ column with an isocratic elution with 15% mobile phase A (water containing 0.025% TFA and 0.5% acetic acid) and 85% mobile phase B at a flow rate of 0.25 mL/min for 6 min using Shimadzu Prominence HPLC system.

The [M+H]^+^ ions were analyzed in the multiple reaction monitoring mode of the ABI/Sciex *API3000* triple quadrupole mass spectrometer equipped with an electrospray ion (ESI) source. Fragmentation occurred at collision gas (argon) pressure of 1.5 mTorr. The observed multiple-reaction monitoring transitions were m/z 175.2→70.0 for ARG, m/z 179.2→71.1 for ^15^N_4_-ARG, m/z 203.2→46.2 for ADMA, m/z 210.4 → 77.3 for D_7_-ADMA, and m/z 181.2→74.2 for IS (^13^C_6_-ARG). Linear standard curves were used to quantify each compound. These included concentration ranges of 10–2000 nM for ARG and ^15^N_4_-ARG, and 2.5–500 nM for ADMA.

### Nitrite/Nitrate assay

The Nitrate/Nitrite Fluorometric Assay Kit (Cayman, MI) was used to determine nitrite concentration in the cell lysates and media samples following the method of Misko et al. [27]. Fluorescence was measured by a SpectraMax Gemini microplate spectrophotometer (Molecular Devices, CA) using excitation/emission wavelengths of 360/430 nm respectively, with a cutoff of 420 nm.

### Statistical analysis

Statistical analyses were performed using Student’s *t*-test, one-way ANOVA, or two-way ANOVA followed by Tukey’s post hoc test, where appropriate. Differences with a p-value of <0.05 were considered statistically significant.

## Results

### Effects of ^15^N_4_-ARG exposure on nitrite production and ADMA fluxes

After EA.hy926 cells were incubated with 100 μM of ^15^N_4_-ARG for 2 hours, nitrite accumulation had significantly increased in ^15^N_4_-ARG-exposed cells compared to control cells (^15^N_4_-ARG: 2.41 ± 0.42 vs. control: 1.78 ± 0.46 nmol/mg, p<0.01).

**Fig 1:**
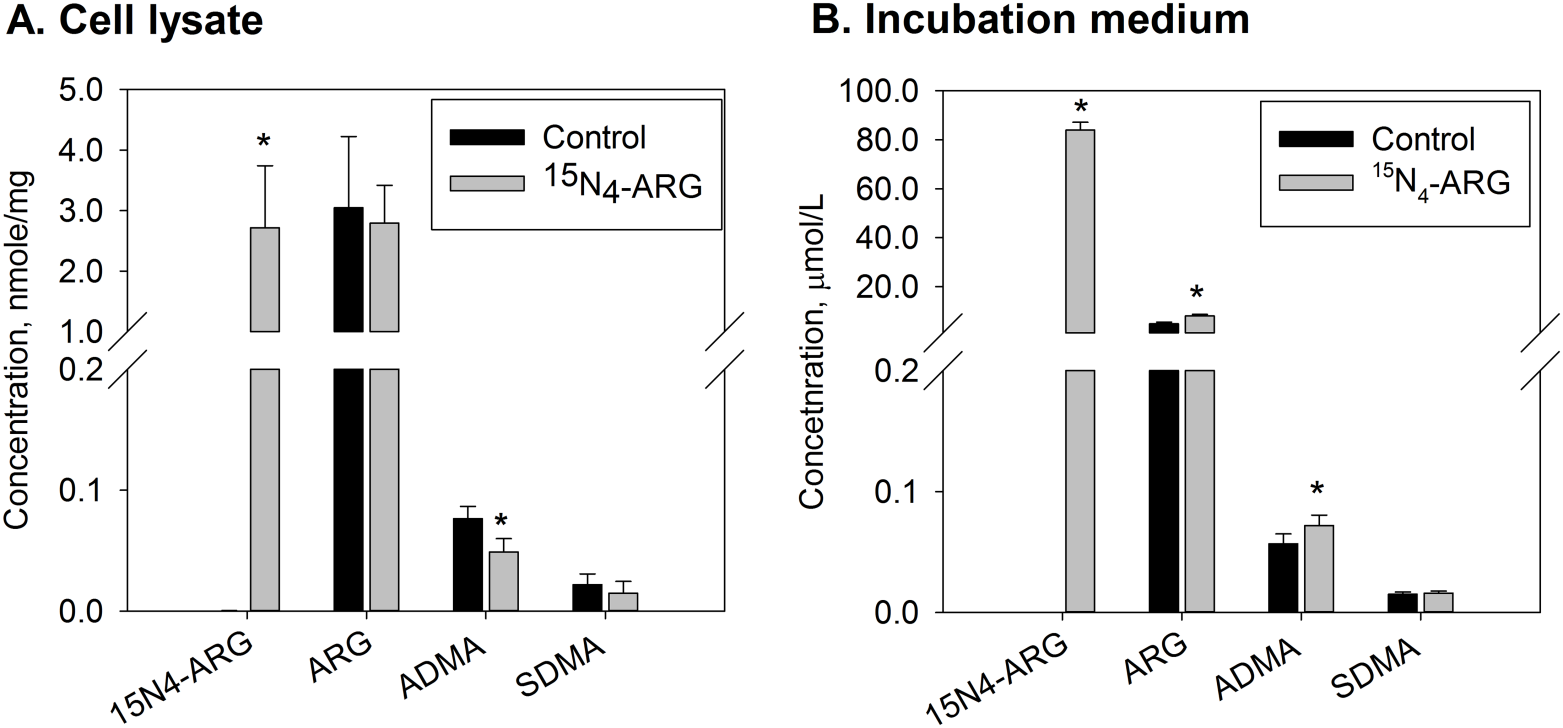
Effects of ^15^N_4_-ARG exposure on the cellular fluxes of ^5^N_4_-ARG, ARG, ADMA, and SDMA in EA.hy926 cells. Concentrations of ^15^N_4_-ARG, ARG, ADMA, and SDMA (A) in the cell lysates and (B) in the incubation media after EA.hy926 cells were incubated with 100 μM of ^15^N_4_-ARG for 2 hours. Data are presented mean ± SD (n = 9). *, p<0.05 vs. control.

The changes in the concentration of endogenous ARG (i.e. ^14^N-ARG), ADMA, and SDMA in cell lysates and incubation medium after exposure to ^15^N_4_-ARG are shown in Fig 1. After incubation, cellular ^15^N_4_-ARG concentration had considerably increased, leading to a large increase in total ARG level (^15^N_4_-ARG + unlabeled ^14^N-ARG) in the cell. While unlabeled, endogenous, cellular ^14^N-ARG and SDMA concentrations did not change considerably, endogenous ADMA concentration had substantially decreased upon ^15^N_4_-ARG exposure (Fig 1A). On the other hand, the concentrations of unlabeled ^14^N-ARG and ADMA in the incubation medium had greatly increased (Fig 1B), while there was no change in SDMA concentration.

### Time course of nitrite production and ADMA fluxes after ^15^N_4_-ARG exposure

**Fig 2:**
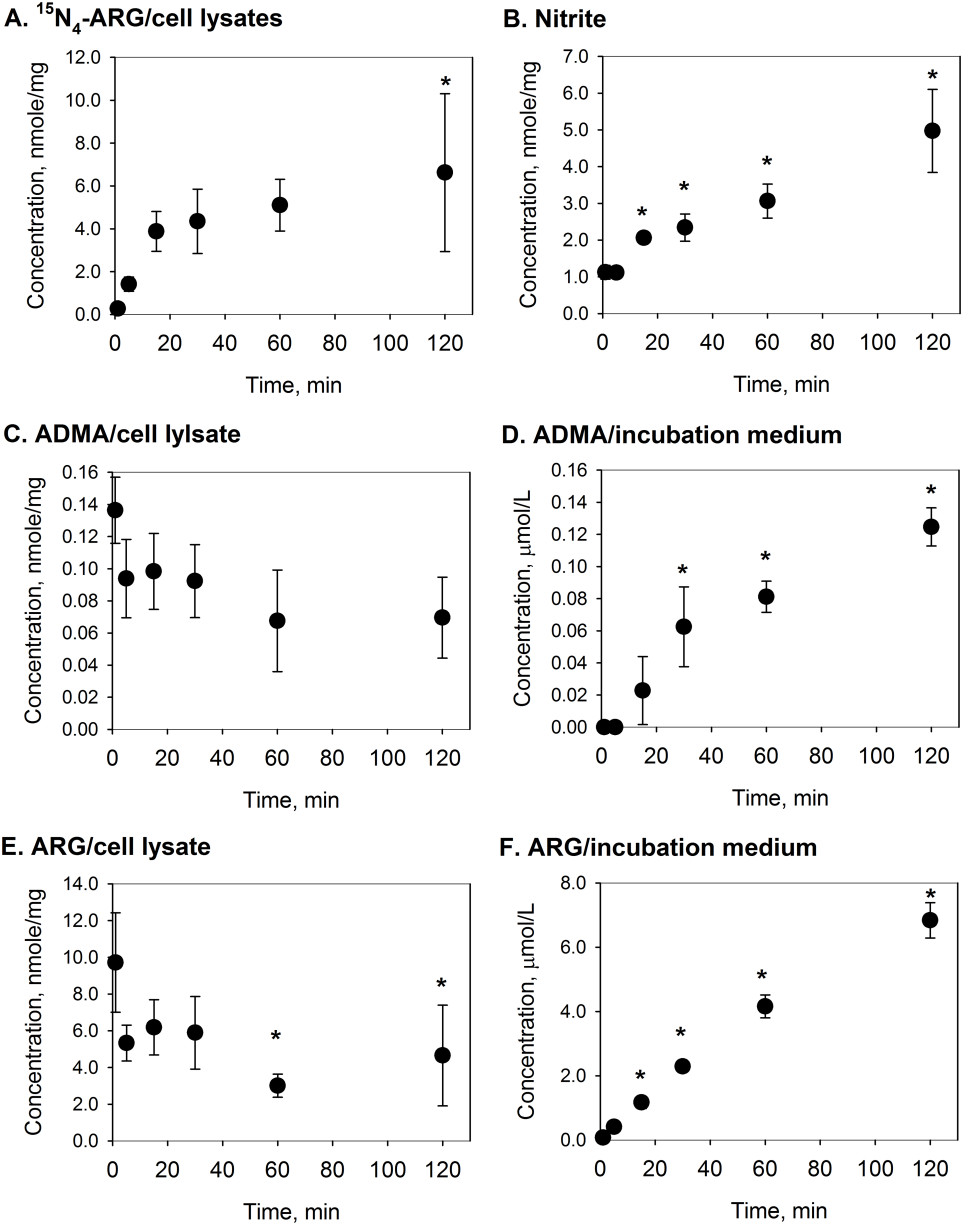
Time courses of NO production and cellular fluxes of ^15^N_4_-ARG, ARG, and ADMA upon ^15^N_4_-ARG exposure. Time courses of (A) ^15^N_4_-ARG uptake, (B) nitrite production, (C) cellular ADMA, (D) extracellular ADMA, (E) cellular ARG, (F) extracellular ARG concentration changes after EA.hy926 cells were exposed to 100 μM ^15^N_4_-ARG. Data are presented mean ± SD (n = 3). *, p<0.05 vs. control, i.e., Time = 0 min.

The changes in nitrite production and ADMA fluxes in parallel to ^15^N_4_-ARG uptake were examined during the period of ^15^N_4_-ARG exposure. Fig 2 shows the time course of ^15^N_4_-ARG uptake and corresponding changes in nitrite accumulation and concentrations of cellular and extracellular ADMA when cells were continuously exposed to 100 μM ^15^N_4_-ARG over 120 minutes. Cellular ^15^N_4_-ARG concentration increased with incubation time, and reached its plateau in 0.5 to 2 hours of exposure (Fig 2A). In parallel to the ^15^N_4_-ARG uptake, nitrite accumulation was found to be substantially increased with time (Fig 2B).

Fig 2 also shows the changes in the concentration of ADMA in the cell lysate (Fig 2C) and incubation medium (Fig 2D) during 120 minutes of exposure to 100 μM ^15^N_4_-ARG. In the cell lysate, ADMA concentration showed a decreasing trend, however, statistical significance could not be demonstrated (p = 0.070, Fig 2C). In the incubation medium, on the other hand, a substantial, gradual increase in ADMA concentration was found over the period of incubation (Fig 2D).

Similar to the observed changes in the ADMA concentration, unlabeled-ARG concentration also had decreased in the cell lysate and had increased in the incubation medium (Fig 2E and 2F). However, since ^15^N_4_-ARG concentration is much greater than ^14^N-ARG concentration in the incubation medium, no measurable change in the combined ^15^N_4_-ARG + ^14^N-ARG concentration was observed. SDMA concentrations were close to the detection limit, and showed no substantial change either in the cell or in the medium (S1 Fig).

### ARG-ADMA interaction in cell membrane fragments

**Fig 3:**
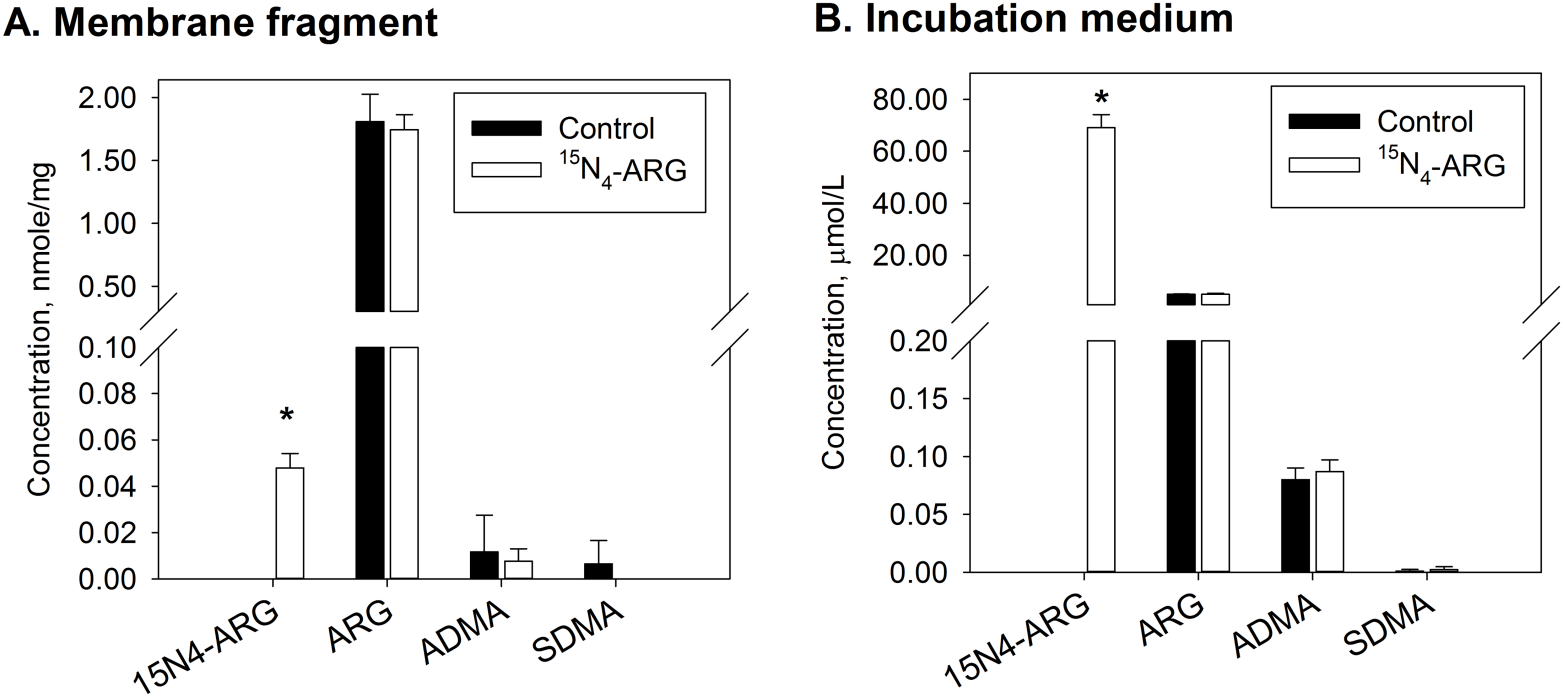
Effects of ^15^N_4_-ARG exposure on the fluxes of ^5^N_4_-ARG, ARG, ADMA, and SDMA in the membrane fragments. Concentrations of ^15^N_4_-ARG, ARG, ADMA, and SDMA (A) in the membrane fragments and (B) in the incubation medium after membrane fragments were incubated with 100 μM of ^15^N_4_-ARG for 2 hours. Data are presented mean ± SD (n = 4). *, p<0.05 vs. control.

Since eNOS is primarily located in the cell membrane, it is of interest to determine whether considerable displacement of ADMA occurs in the membrane upon addition of extracellular ARG. Fig 3 shows the concentrations of ^15^N_4_-ARG along with other ARG-related, endogenous amino acids in the membrane fragments (Fig 3A) and the external solution (Fig 3B) after membrane fragments were incubated with 100 μM ^15^N_4_-ARG for 1 hour. After ^15^N_4_-ARG exposure, a large quantity of ^15^N_4_-ARG was found to be incorporated into the membrane fragments, while no substantial change in endogenous ARG, ADMA, and SDMA was observed (Fig 3A). The concentrations of these amino acids in the incubation medium were not altered by the increased ^15^N_4_-exposure (Fig 3B).

### Trans-stimulation of cellular ARG and ADMA efflux

**Fig 4:**
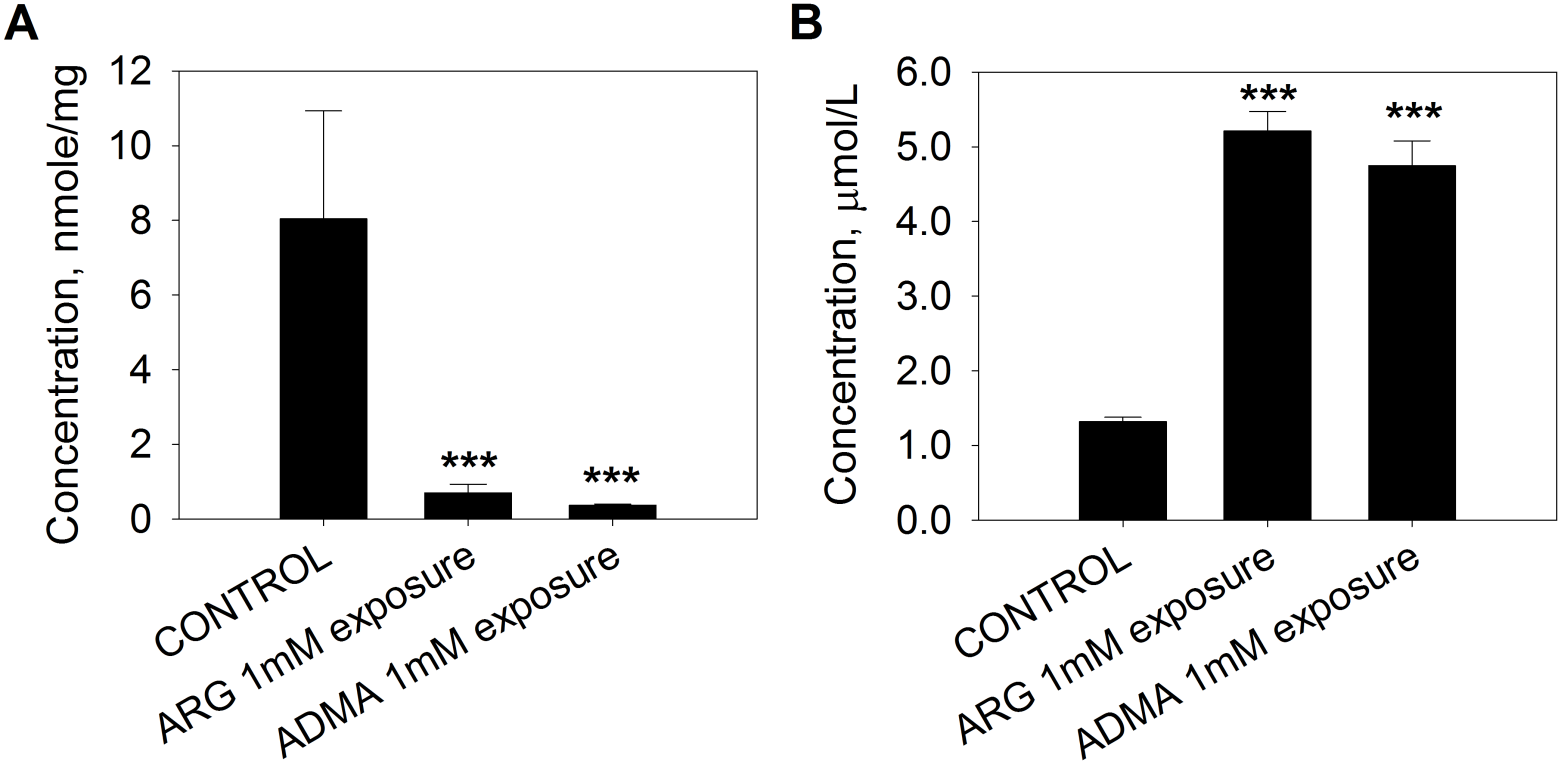
Trans-stimulated ^15^N_4_-ARG efflux by extracellular ARG and ADMA exposure. EA.hy926 cells were pre-incubated with 100 μM ^15^N_4_-ARG, washed and then exposed to 1 mM ARG or 1 mM ADMA for 1 hour. ^15^N_4_-ARG concentrations were determined (A) in the cell lysate and (B) in the incubation medium. Data are presented mean ± SD (n = 4). ***, p<0.001 vs. control.

To examine whether extracellular ARG exposure indeed, stimulates cellular ARG and ADMA efflux by trans-stimulation, the influence of extracellular ARG and ADMA on the efflux of stable isotope-labeled ARG (^15^N_4_-ARG) and ADMA (D_7_-ADMA) was determined. Fig 4 shows that when cells were preincubated with 100 μM of ^15^N_4_-ARG for 1 hour, followed by ARG (1 mM) or ADMA (1 mM) exposure, cellular ^15^N_4_-ARG was greatly depleted (Fig 4A) while increases in the level of extracellular ^15^N_4_-ARG were observed compared to those of the control (Fig 4B). These results indicate that cellular efflux of ^15^N_4_-ARG occurred in spite of either an unfavorable ARG concentration gradient, or in the presence of an extracellular concentration of 1 mM ADMA.

**Fig 5:**
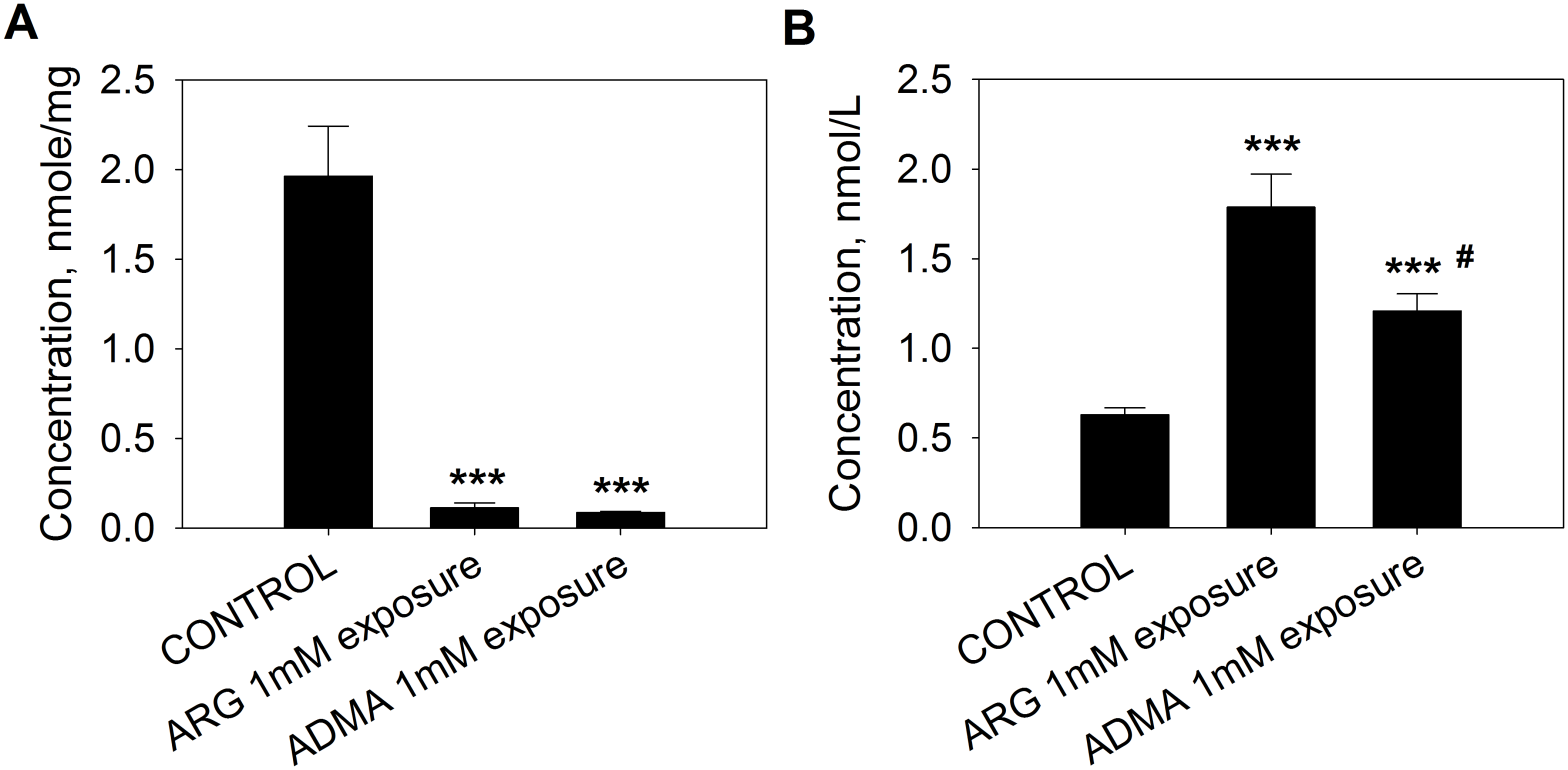
Trans-stimulated D_7_-ADMA efflux by extracellular ARG and ADMA exposure. EA.hy926 cells were pre-incubated with 10 μM D_7_-ADMA, washed and then exposed to 1 mM ARG or 1 mM ADMA for 1 hour. D_7_-ADMA concentrations were determined (A) in the cell lysate and (B) in the incubation medium. Data are presented mean ± SD (n = 4). ***, p<0.001 vs. control; ^#^, p<0.05 vs. ARG 1 mM.

To demonstrate trans-stimulation of ADMA efflux, cells were preincubated with 10 μM of D_7_-ADMA for 1 hour, and then exposed to extracellular ARG (1 mM) or ADMA (1 mM). The addition of ARG or ADMA to the extracellular space at a high concentration led to a profound decrease in cellular D_7_-ADMA level (Fig 5A), and an increase in extracellular D_7_-ADMA level (Fig 5B) compared with untreated cells.

### Effect of ARG exposure on D_7_-ADMA metabolism in whole cells

To examine whether ARG exposure affects cellular ADMA metabolism, the diminution of D_7_-ADMA was monitored by using EA.hy926 cells, which were preincubated with 100 μM D_7_-ADMA prior to ARG exposure. Table 1 shows that after the cells were exposed to 100 μM ARG for 2 hours, the levels of the remaining D_7_-ADMA in the whole system was considerably greater than those in the control treated cells. The levels of endogenous ADMA were higher after ARG exposure, but no statistical difference was found. Total ADMA (labeled and unlabeled) levels also had increased after ARG exposure. As expected, after exposure to ARG, cells exhibited increased ARG levels. SDMA concentrations were not largely affected by ARG exposure. Protein concentrations were not considerably different between the two groups.

**Table 1:** Effects of ARG exposure on D_7_-ADMA metabolism.

		Control	ARG Exposure
D_7_-ADMA	nmol/mg	26.5 ± 5.8	33.6 ± 3.8 *
ADMA	nmol/mg	2.71 ± 0.30	3.29 ± 0.58
Total ADMA	nmol/mg	29.2 ± 5.5	36.9 ± 4.2 *
ARG	nmol/mg	119 ± 12	405 ± 15 *
SDMA	nmol/mg	0.640 ± 0.117	0.789 ± 0.143
Protein	μg/mL	696 ± 45	694 ± 20

EA.hy926 cells were pre-incubated with 100 μM D_7_-ADMA, washed, and then exposed to 100 μM ARG for 2 hours. The concentrations in the cell lysate and in the incubation medium which were measured by the LC-MS/MS assay were converted to amounts (in nmoles) and combined to estimate the contents in the whole system (in nmol/mg protein) based on the protein contents in the sample.

Data are presented mean ± SD (n = 6).

*, p<0.05 vs. Control.

### Effect of ARG exposure on D_7_-ADMA metabolism in cell lysates

**Fig 6:**
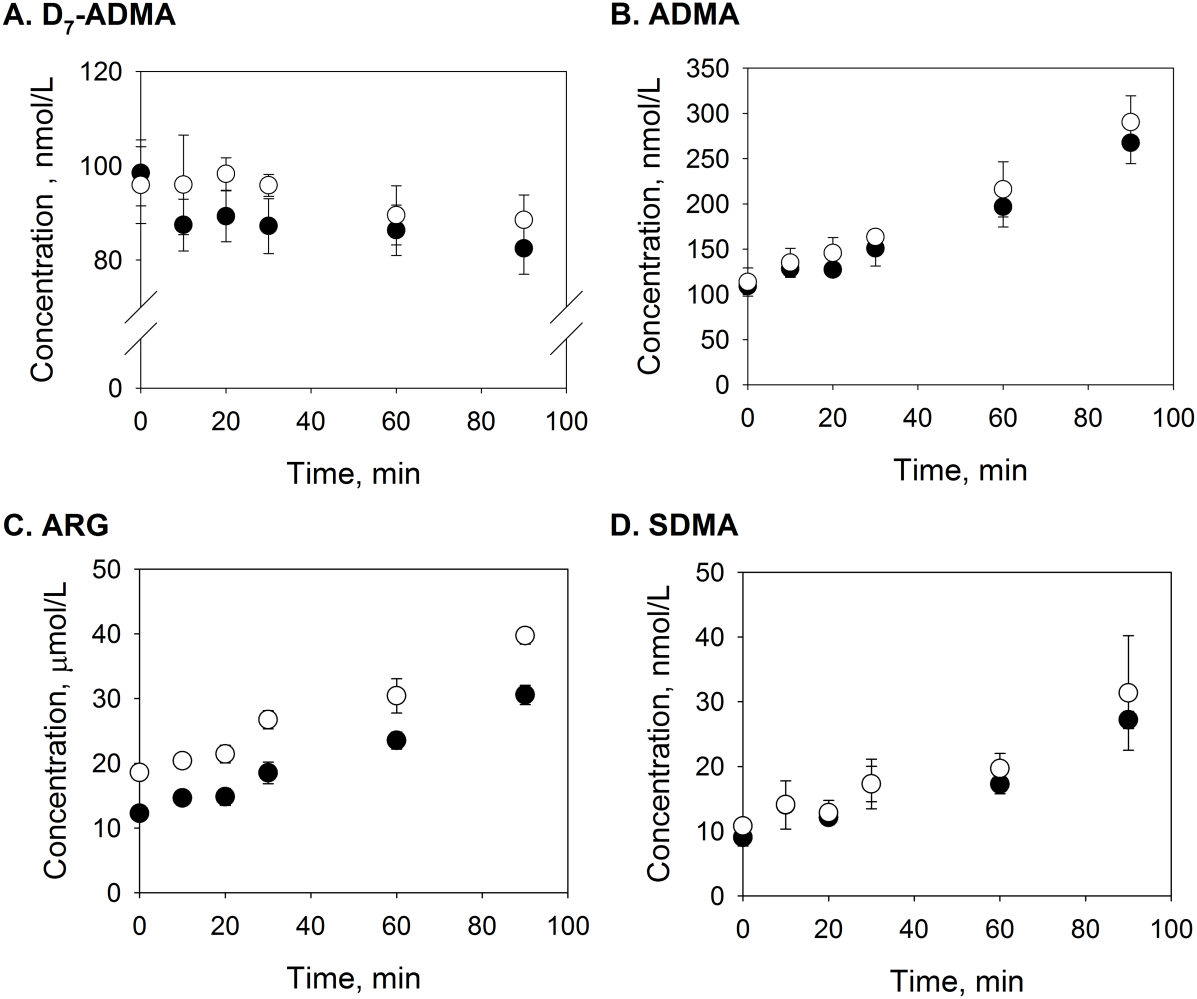
Effects of ARG exposure on the fluxes of D_7_-ADMA, ADMA, ARG, and SDMA in the cell lysates. Concentration changes of (A) D_7_-ADMA, (B) ADMA, (C) ARG, and (D) SDMA in the cell lysates after cell lysates were incubated 0.1 μM D_7_-ADMA alone (closed circles) or 0.1 μM D_7_-ADMA and 5 μM ARG (open circles) over 2 hours. Data are presented mean ± SD (n = 3).

To eliminate the influence of cellular transport, the effects of ARG exposure on D_7_-ADMA metabolism was also examined using endothelial cell lysates. After the EA.hy926 cells were lysed, the cell lysates were exposed to 0.1 μM D_7_-ADMA or 0.1 μM D_7_-ADMA + 5 μM ARG. Fig 6A shows D_7_-ADMA concentrations in cell lysates after incubation in the presence of 5 μM ARG vs. control. Two-way ANOVA showed that these concentrations were affected by time (p = 0.046) and treatment (i.e., ARG vs. control, p = 0.014). Although D_7_-ADMA concentrations appeared higher after ARG treatment, post-hoc analysis did not reveal any marked difference when they were compared to the corresponding data points in the control group. Endogenous ADMA levels (Fig 6B) had increased with exposure time, and the endogenous ADMA concentration was also, affected by the addition of 5 μM ARG, as determined by the two-way ANOVA test.

In these cell-lysate samples, D_7_-ADMA and D_7_-ADMA+ARG incubation also led to apparent increase in ARG and SDMA concentrations over time (Fig 6C and 6D). However, post hoc analysis showed that the addition of 5 μM ARG produced statistical differences only in the measured ARG concentrations in cell lysates (which was expected), and not in the SDMA level.

## Discussion

In this study, the effects of ^15^N_4_-ARG exposure on the fluxes of various cellular compounds, including ARG, ADMA, and SDMA were examined. By using stable isotope-labeled ARG, i.e., ^15^N_4_-ARG, as the added amino acid, we were able to monitor the fate of its cellular uptake vs. the efflux of the endogenous pool of ADMA and unlabeled ARG, i.e., ^14^N-ARG.

Our results showed that nitrite accumulation upon ^15^N_4_-ARG uptake was accompanied by increases in endogenous ^14^N-ARG and ADMA concentrations in the extracellular fluid, suggesting that ^15^N_4_-ARG exposure stimulated the efflux of these amino acids from the cell. By using the stable isotope-labeled analogues, ^15^N_4_-ARG and D_7_-ADMA (Figs 4 and 5), we confirmed the trans-stimulation of the efflux of these amino acids by each other in EA.hy926 cells. The trans-stimulated cellular effluxes of ^15^N_4_-ARG and D_7_-ADMA were also observed in another cell line, human umbilical vein endothelial cells (S2 and S3 Figs). This phenomenon did not appear to affect cellular SDMA fluxes greatly, which were largely unaffected by ^15^N_4_-ARG exposure.

ADMA and SDMA share similar affinity to the ARG transporter, CAT-1 [11, 12], and therefore, it is likely that this transporter may be involved in mediating the observed transport events. The enhanced efflux of endogenous ARG and ADMA due to extracellular ^15^N_4_-ARG exposure is consistent with the trans-stimulation of system y+ transporters (which include CAT-1) that mediate the transmembrane exchange of extracellular and intracellular cationic amino acids. Our observations are consistent with the findings that cationic and neutral amino acids limit ARG uptake as well as stimulate cellular ARG efflux from EA.hy926 cells [10], which were reversed by ARG incubation.

Because ADMA is an endogenous inhibitor of NOS, its interaction with ARG has been proposed to contribute to the mechanism of the “ARG paradox.” This phenomenon exists because the intracellular concentration of endogenous ARG far exceeds the Km of NOS for ARG, and hence, minor increases in ARG concentrations resulting from its supplementation would not increase enzyme activity. ADMA involvement can potentially explain this phenomenon because its presence raises the apparent Km of eNOS and hence, supplemented exogenous ARG displaces ADMA from the enzyme and restores NO production [6].

However, it is recognized that the endogenous plasma ADMA concentrations, of about 0.5 μM may be too small to exert an effect that is sufficient to explain the ARG paradox. In various pathological conditions, such as chronic renal failure, hypercholesterolemia, pre-eclampsia, hypertension, type 2 diabetes mellitus, pulmonary hypertension, and coronary artery disease, and so on, the plasma concentrations of ADMA are elevated because of its impaired metabolism by dimethylarginine dimethylaminohydrolase [17–20]. However, even in these diseases, the circulating ADMA concentration in plasma may be still too low (0.4–6.0 μM).

The results of this study demonstrated that interactions between ARG and ADMA also occurred in relation to cellular transport. Therefore, ARG supplementation may not only displace ADMA from the eNOS enzyme site, but also reduce intracellular ADMA concentration by stimulating its efflux. It is currently unknown how the latter affects the net concentration of ADMA at the eNOS binding site. However, no considerable increase in ADMA displacement in the cell membrane due to exposure to high concentrations of ^15^N_4_-ARG was observed, when membrane fragments were studied separately (Fig 3), suggesting that most of the displaced ADMA have an intracellular origin. The amount of membrane-bound ADMA was low, near the detection limit of our assay. This may have resulted from the preparation procedure, which involved multiple ultracentrifugation steps. The ARG/ADMA ratio observed in our membrane preparation was 91.7 ± 41.8, compared to an intracellular ratio of 72.5 ± 8.9. In bovine aortic endothelial cells, the reported intracellular concentrations of ARG and ADMA were 151 ± 34 and 3.6 ± 1.0 μM, respectively, yielding a ratio of approximately 42 [5]. The mean ARG/ADMA ratio in plasma of healthy subjects ranged from 132–227 [7].

Changes in cellular ADMA concentration may not be restricted to those involving molecular transport. Studies have shown that ARG may inhibit the metabolism of ADMA in HepG2 cells [21], and ARG is a competitive inhibitor of the ADMA-metabolizing enzyme, DDAH in the rat kidney [28]. Since it is not known how ARG exposure might affect the synthesis and metabolism of endogenous ADMA, we employed D_7_-ADMA, which is not present endogenously, to examine the effect of ARG exposure on its diminution, both in whole cells (Table 1) and in cell lysates (Fig 6). In the study with cell lysates, an ARG concentration of 5 μM was chosen based on the total intracellular ARG concentration that was observed after cells were exposed to 100 μM ^15^N_4_-ARG for 2 hours. Our results revealed that, in our experimental system, the ARG-induced inhibition of D_7_-ADMA metabolism was statistically significant, though the effect was quite small. Nevertheless, these data demonstrated that ARG exposure could also affect the metabolism of endogenous ADMA. Similar results were obtained in the experiments using HUVEC (S1 Table and S4 Fig).

In the study with cell lysates, we observed an increase in the accumulation of unlabeled ADMA, ARG, and SDMA with incubation time. These results are explained by the rapid proteolysis of methylated proteins in vitro. When rat-kidney homogenate was incubated in vitro for 60 min, the release of free ADMA and SDMA associated with the proteolysis of methylated proteins as well as the release of protein-bound ARG were observed [29]. The concomitant increase in endogenous ADMA level during the incubation period might therefore have dampened the effect of the added ARG on D_7_-ADMA metabolism.

## Conclusion

These results indicated that added ^15^N_4_-ARG exposure to EA.hy926 cells expectedly enhanced nitrite accumulation, however, its uptake also, induced changes in the fluxes of ADMA. Cellular metabolism of ADMA was also inhibited by ARG exposure. Therefore, ARG supplementation may alter the cellular availability of ADMA via multiple mechanisms, which may affect its overall inhibitory effect on eNOS.

## Supporting information

S1 Fig Time course of the cellular flux of SDMA upon ^15^N_4_-ARG exposure in EA.hy926 cells. Time courses of (A) cellular SDMA, (B) extracellular SDMA concentration changes after EA.hy926 cells were exposed to 100 μM ^15^N_4_-ARG. Data are presented mean ± SD (n = 3). *, p<0.05 vs. control, i.e., Time = 0 min.
(DOCX)

S2 Fig Trans-stimulated ^15^N_4_-ARG efflux by extracellular ARG and ADMA exposure in HUVECs. HUVEC cells were pre-incubated with 500 μM ^15^N_4_-ARG, washed and then exposed to 1 mM ARG or 1 mM ADMA for 1 hour. ^15^N_4_-ARG concentrations were determined (A) in the cell lysate and (B) in the incubation medium. Data are presented mean ± SD (n = 4). *, p<0.05 vs. control.
(DOCX)

S3 Fig Trans-stimulated D_7_-ADMA efflux by extracellular ARG and ADMA exposure in HUVECs. HUVEC cells were pre-incubated with 500 μM D_7_-ADMA, washed and then exposed to 1 mM ARG or 1 mM ADMA for 1 hour. D_7_-ADMA concentrations were determined (A) in the cell lysate and (B) in the incubation medium. Data are presented mean ± SD (n = 3). *, p<0.05 vs. control.
(DOCX)

S4 Fig Effects of ARG exposure on the fluxes of D_7_-ADMA, ADMA, ARG, and SDMA in the HUVEC cell lysates. Concentration changes of (A) D_7_-ADMA, (B) ADMA, (C) ARG, and (D) SDMA in the cell lysates after cell lysates were incubated 5 μM D_7_-ADMA alone (closed circles) or 5 μM D_7_-ADMA and 5 μM ARG (open circles) for 90 min. Data are presented mean ± SD (n = 3).
(DOCX)

S1 Table Effects of ARG exposure on D7-ADMA metabolism in HUVEC cells. Cells were pre-incubated with 500 μM D_7_-ADMA, washed, and then exposed to 1 mM ARG or 1 mM ADMA for 1 hour. The concentrations in the cell lysate and in the incubation medium which were measured by the LC-MS/MS assay were converted to amounts (in nmoles) and combined to estimate the contents in the whole system (in nmol/mg protein) based on the protein contents in the sample. Data are presented mean ± SD (n = 3). *, p<0.05 vs. Control.
(DOCX)
