# Supplementary material for: Cellular interactions between L-arginine and asymmetric dimethylarginine: Transport and metabolism
Source: PLoS One. 2017 May 31;12(5):e0178710. doi: 10.1371/journal.pone.0178710 (PMC5451097; doi:10.1371/journal.pone.0178710)
Supplement: S1 Fig — Time courses of (A) cellular SDMA, (B) extracellular SDMA concentration changes after EA.hy926 cells were exposed to 100 μM 15N4-ARG. Data are presented mean ± SD (n = 3). *, p<0.05 vs. control, i.e., Time = 0 min. (DOCX) [file pone.0178710.s001.docx]

**S1 Fig. Time course of the cellular flux of SDMA upon ^15^N_4_-ARG exposure in EA.hy926 cells.**

Time courses of (A) cellular SDMA, (B) extracellular SDMA concentration changes after EA.hy926 cells were exposed to 100 μM ^15^N_4_-ARG. Data are presented mean ± SD (n=3). *, p<0.05 vs. control, i.e., Time = 0 min.
