# Supplementary material for: Cellular interactions between L-arginine and asymmetric dimethylarginine: Transport and metabolism
Source: PLoS One. 2017 May 31;12(5):e0178710. doi: 10.1371/journal.pone.0178710 (PMC5451097; doi:10.1371/journal.pone.0178710)
Supplement: S2 Fig — HUVEC cells were pre-incubated with 500 μM 15N4-ARG, washed and then exposed to 1 mM ARG or 1 mM ADMA for 1 hour. 15N4-ARG concentrations were determined (A) in the cell lysate and (B) in the incubation medium. Data are presented mean ± SD (n = 4). *, p<0.05 vs. control. (DOCX) [file pone.0178710.s002.docx]

**S2 Fig. Trans-stimulated ^15^N_4_-ARG efflux by extracellular ARG and ADMA exposure in HUVECs**.

HUVEC cells were pre-incubated with 500 μM ^15^N_4_-ARG, washed and then exposed to 1 mM ARG or 1 mM ADMA for 1 hour. ^15^N_4_-ARG concentrations were determined (A) in the cell lysate and (B) in the incubation medium. Data are presented mean ± SD (n=4). *, p<0.05 vs. control.
