# Supplementary material for: Cellular interactions between L-arginine and asymmetric dimethylarginine: Transport and metabolism
Source: PLoS One. 2017 May 31;12(5):e0178710. doi: 10.1371/journal.pone.0178710 (PMC5451097; doi:10.1371/journal.pone.0178710)
Supplement: S4 Fig — Concentration changes of (A) D7-ADMA, (B) ADMA, (C) ARG, and (D) SDMA in the cell lysates after cell lysates were incubated 5 μM D7-ADMA alone (closed circles) or 5 μM D7-ADMA and 5 μM ARG (open circles) for 90 min. Data are presented mean ± SD (n = 3). (DOCX) [file pone.0178710.s004.docx]

**S4 Fig.** **Effects of ARG exposure on the fluxes of D_7_-ADMA, ADMA, ARG, and SDMA in the HUVEC cell lysates.**

Concentration changes of (A) D_7_-ADMA, (B) ADMA, (C) ARG, and (D) SDMA in the cell lysates after cell lysates were incubated 5 µM D_7_-ADMA alone (closed circles) or 5 µM D_7_-ADMA and 5 µM ARG (open circles) for 90 min. Data are presented mean ± SD (n=3).
