# Supplementary material for: Cellular interactions between L-arginine and asymmetric dimethylarginine: Transport and metabolism
Source: PLoS One. 2017 May 31;12(5):e0178710. doi: 10.1371/journal.pone.0178710 (PMC5451097; doi:10.1371/journal.pone.0178710)
Supplement: S1 Table — Cells were pre-incubated with 500 μM D7-ADMA, washed, and then exposed to 1 mM ARG or 1 mM ADMA for 1 hour. The concentrations in the cell lysate and in the incubation medium which were measured by the LC-MS/MS assay were converted to amounts (in nmoles) and combined to estimate the contents in the whole system (in nmol/mg protein) based on the protein contents in the sample. Data are presented mean ± SD (n = 3). *, p<0.05 vs. Control. (DOCX) [file pone.0178710.s005.docx]

**S1 Table. Effects of ARG exposure on D_7_-ADMA metabolism in HUVEC cells.**

Cells were pre-incubated with 500 μM D_7_-ADMA, washed, and then exposed to 1 mM ARG or 1 mM ADMA for 1 hour. The concentrations in the cell lysate and in the incubation medium which were measured by the LC-MS/MS assay were converted to amounts (in nmoles) and combined to estimate the contents in the whole system (in nmol/mg protein) based on the protein contents in the sample. Data are presented mean ± SD (n=3). *, p<0.05 vs. Control.

|  |  | Control | ARG Exposure |
| --- | --- | --- | --- |
| D_7_-ADMA | nmol/mg | 42.7 ± 9.4 | 64.6 ± 7.8 * |
| ADMA | nmol/mg | 0.86 ± 1.06 | 0.29 ± 0.10 |
| Total ADMA | nmol/mg | 43.5 ± 8.9 | 64.9 ± 7.8 * |
| ARG | nmol/mg | 16.8 ± 3.2 | 94615 ± 6980 * |
| SDMA | nmol/mg | ND | ND |
| Protein | mg/mL | 0.38 ± 0.02 | 0.27 ± 0.03 * |
